# Integrative Analysis of GEO Datasets and Mendelian Randomization Reveals a Potential Role ofISOC1 in Renal Cell Carcinoma

**DOI:** 10.7150/jca.118622

**Published:** 2025-10-10

**Authors:** Jingsong Wang, Yunxun Liu, Zhiwei Yan, Qianxue Lu, Jun Jian, Xiuheng Liu, Zhiyuan Chen, Qingyuan Zheng, Shanshan Wan, Lei Wang

**Affiliations:** 1 Department of Urology, Renmin Hospital of Wuhan University, Wuhan, Hubei, China.; 2 Institute of Urologic Disease, Renmin Hospital of Wuhan University, Wuhan, Hubei, China.; 3 Department of Ophthalmology, Renmin Hospital of Wuhan University, Wuhan, Hubei, China.

**Keywords:** renal cell carcinoma, eQTL, pQTL, Mendelian randomization

## Abstract

**Background:** Renal cell carcinoma (RCC) is a leading malignancy of the urinary system, with clear cell RCC (ccRCC) being the most prevalent subtype. Despite advances in treatment, the prognosis of advanced RCC remains poor, and the molecular mechanisms underlying its pathogenesis are not fully understood.

**Methods:** This study utilized multiple renal cancer cohorts from the Gene Expression Omnibus (GEO) database to identify differentially expressed genes (DEGs). By integrating Mendelian randomization (MR) analyses of expression quantitative trait loci (eQTL) and protein quantitative trait loci (pQTL), we investigated causal associations between candidate genes and RCC. Immune infiltration, drug sensitivity, and survival analyses were performed to further explore functional significance. In vitro experiments validated the biological role ofISOC1 in RCC progression.

**Results:** We focused on ISOC1, a gene previously implicated in other malignancies but not well studied in RCC. Through integrative MR analysis, we identified ISOC1 as a novel RCC-associated gene, with potential tumor-suppressive functions in this specific context. ISOC1 expression was significantly linked to tumor immune infiltration, drug sensitivity, and patient prognosis. Functional assays demonstrated that ISOC1 knockdown promoted RCC cell proliferation, migration, and invasion.

**Conclusions:** ISOC1 plays a critical role in RCC progression and may act as a tumor suppressor. These findings highlight ISOC1 as a potential biomarker for prognosis and a promising target for therapeutic intervention in RCC. Moreover, this study underscores the utility of MR-based integrative analyses in uncovering novel molecular mechanisms and therapeutic targets for cancer.

## Introduction

Renal cell carcinoma (RCC) is a major malignancy of the urinary system and represents the most common type of kidney malignant tumor. RCC is classified into several subtypes based on histological features, with clear cell renal cell carcinoma (ccRCC) being the most prevalent, accounting for approximately 70-80% of all RCC cases^1^,^2^. In addition to ccRCC, other notable subtypes include papillary renal cell carcinoma (Papillary RCC) and chromophobe renal cell carcinoma (Chromophobe RCC)^3^. The exact etiology of RCC remains incompletely understood, but several risk factors have been identified, including smoking, obesity, hypertension, and certain genetic conditions. RCC is typically asymptomatic in its early stages, and many patients are diagnosed only when the tumor has reached a considerable size or has metastasized^4^. For early-stage RCC with smaller tumors, partial nephrectomy can yield favorable outcomes^5^. However, in cases of larger tumors, radical nephrectomy is often required, placing significant psychological and financial burdens on patients. While surgical intervention for localized RCC can offer a relatively favorable prognosis, the prognosis for advanced or metastatic disease remains poor^6^. Despite recent advances in targeted therapies and immunotherapy, the risk of recurrence and metastasis remains high^7^,^8^. Due to its insidious onset and propensity for recurrence and metastasis, RCC treatment continues to pose significant challenges. Enhancing early detection methods and identifying new molecular targets remain key focuses of ongoing research.

Mendelian Randomization (MR) is a sophisticated methodological approach that employs genetic variants as instrumental variables to investigate causal relationships. It is extensively utilized in epidemiological research, particularly for elucidating the causal links between environmental factors, lifestyle behaviors, biomarkers, and diseases^9^. By capitalizing on the random inheritance of genetic variation, MR effectively mitigates confounding and reverse causation, common limitations of traditional observational studies, thereby offering more robust and reliable causal inferences^10^. eQTL (expression quantitative trait locus) refers to loci associated with variations in gene expression levels. By examining the relationship between genetic variants, such as single nucleotide polymorphisms (SNPs), and gene expression, eQTL studies aim to identify genetic variations that influence transcriptional activity, thereby impacting disease susceptibility or phenotypic traits^11^-^13^. Employing eQTL in Mendelian randomization analysis allows for a deeper understanding of how genetic variations can alter gene expression to modulate complex traits or disease predisposition^14^. In contrast, pQTL (protein quantitative trait locus) pertains to loci associated with variations in protein levels. Mendelian randomization leveraging pQTL primarily focuses on how genetic variations affect protein expression, post-translational modifications, or protein functionality^15^. By investigating the associations between genetic variations and protein expression levels, pQTL studies provide valuable insights into how genetic factors influence protein quantity or function, further elucidating the molecular mechanisms underlying diseases^16^,^17^. While eQTL and pQTL can be studied separately, they are often interconnected. Integrating both eQTL and pQTL in Mendelian randomization analysis offers a powerful approach to identify potential targets and mechanisms driving disease pathogenesis.

In our study, we initially identified differentially expressed genes (DEGs) through analysis of multiple renal cancer cohorts from the Gene Expression Omnibus (GEO) database. Subsequently, by integrating eQTL and pQTL Mendelian randomization analyses, we uncovered a robust genetic association between isochorismatase domain-containing protein 1(ISOC1) and renal cancer. Among the genes identified as significantly associated with renal cell carcinoma through eQTL MR analysis, ISOC1 was the only gene that also demonstrated a strong causal association at the protein level, as revealed by pQTL MR analysis. This dual-layer evidence suggested that ISOC1 may play a crucial role in RCC pathogenesis and warranted further investigation through functional experiments. This finding was further substantiated through immune infiltration analysis, drug sensitivity profiling, and survival prognosis analysis. Finally, in vitro experiments confirmed that the downregulation of ISOC1 significantly enhanced the proliferation, migration, and invasion of RCC cells.

## Materials and Methods

### Data collection and identification of DEGs

We downloaded transcriptomic data of five renal cancer cohorts from the GEO database and performed data correction separately. Four of these datasets were merged, followed by principal component analysis (PCA) for data normalization and batch effect correction. Differential expression analysis was performed on the merged data using the “limma” R package, applying a significance threshold of a corrected P-value < 0.05 and an absolute log fold change (logFC) > 0.585 to identify DEGs. Volcano plots and heatmaps of the DEGs were generated using the “pheatmap” R package."

### eQTL and pQTL summary statistics

We retrieved peripheral blood eQTL data from 5,311 European individuals in the Genome-Wide Association Study (GWAS) database (https://gwas.mrcieu.ac.uk/)^18^ and employed the “TwoSampleMR” R package to identify SNPs with strong associations (p < 5 × 10⁻⁸) as instrumental variables. To minimize potential confounding, we set the linkage disequilibrium (LD) threshold at r2 < 0.001 and a clustering distance of 10,000 kb. Additionally, we applied a filter of F-statistic > 10 to remove SNPs with weak associations or insufficient phenotypic variance explanation. Our PQTL data originated from the study by Ferkingstad, E. et al.^19^, which examined plasma protein levels of 35,559 analytes in 4,907 Icelandic individuals. We obtained the raw PQTL data from the website (https://www.decode.com/summarydata/) and conducted association analysis and linkage disequilibrium correction using the same methodology employed for the previously described eQTL analysis.

### Mendelian randomization (MR) analysis

The outcome data for this study is derived from the Finnish database (https://www.finngen.fi/en/access_results) with the dataset ID “finn-b- C3_KIDNEY_NOTRENALPELVIS_EXALLC”. It encompasses 971 kidney cancer cases and 174,006 controls from the European population, comprising a total of 16,380,308 SNPs.

We performed a two-sample MR analysis to assess the causal relationship between the exposure and the outcome based on the “TwoSampleMR” R package. The inverse-variance weighted (IVW) method was used to combine the estimates, and sensitivity analyses were performed using the MR- Egger and weighted median methods to evaluate the potential presence of pleiotropy. To assess the robustness of the results, we conducted several sensitivity analyses, including leave-one-out analysis to identify influential variants. Genes identified as positive in the Mendelian randomization analysis were selected based on the following criteria: (1) the p-value from the inverse variance weighted (IVW) method was less than 0.05; (2) the direction of the odds ratios (OR) was consistent across all five analysis methods; and (3) the p-value from the pleiotropy test was greater than 0.05, indicating no significant pleiotropy. To assess the consistency of genetic instrument effects, we also performed heterogeneity tests using Cochran's Q test. Genetic instruments with a p-value from the Q test greater than 0.05 were considered to have consistent effects across the study samples, and no significant heterogeneity was observed. Finally, to identify potential causal genes, we performed a cross-validation analysis between the genes identified through Mendelian randomization and DEGs.

### Gene function enrichment analysis

Gene Ontology (GO) enrichment analysis was performed to categorize the biological functions of the intersection genes into three categories: biological processes (BP), molecular functions (MF), and cellular components (CC). We utilized the “clusterProfiler” R package to assess the enrichment of GO terms, with a significance threshold of p-value < 0.05. Additionally, Kyoto Encyclopedia of Genes and Genomes (KEGG) pathway enrichment analysis was conducted to identify relevant pathways, with candidate genes mapped to known KEGG pathways and statistical significance evaluated. Gene Set Enrichment Analysis (GSEA) was performed to investigate the biological pathways associated with the high and low expression of the gene “ISOC1”. Pathways with an FDR < 0.25 were considered significantly enriched. GSEA results were visualized, and additional enrichment analyses were conducted using the “clusterProfiler” R package.

### Immune cell infiltration analysis

To investigate the immune cell infiltration associated with ISOC1 expression in the TCGA-KIRC cohort, we used the CIBERSORT algorithm, which estimates the relative abundance of 22 immune cell types from gene expression profiles. All RCC samples were stratified into two groups based on the median expression level ofISOC1. CIBERSORT was applied to analyze immune cell infiltration in each group, and the relative abundance of each immune cell type was compared between the two groups to assess the impact ofISOC1 expression on the tumor immune microenvironment.

### Drug sensitivity analysis

Drug sensitivity was evaluated by estimating the half maximal inhibitory concentration (IC50) values for a variety of chemotherapeutic agents using the “oncePredict” R package. The IC50 represents the concentration of a drug required to inhibit cell viability by 50%. The IC50 values for each group were calculated and compared to assess the differential drug sensitivity associated with ISOC1 expression.

### Cell culture and treatment

The human ccRCC cell lines 786-O, 769-P and normal kidney tubular epithelial cell HK-2 used in this study were obtained from the American-Type Culture Collection (ATCC) and authenticated by STR profiling to ensure the correctness of the cell line. The 786-O and 769-P cell lines were cultured in RPMI 1640 (Cytiva, Logan Utah, USA) supplemented with10% fetal bovine serum media (FBS) (GIBCO, Grand Island, NY, USA), while the HK-2 cell line was cultured in Ham's F-12K/10% fetal bovine serum media. The cells were maintained in a humidified incubator at 37°C with 5% CO2 and passaged at a ratio of 1:3 using 0.25% Trypsin-EDTA when confluence reached 80-90%.

Small interfering RNA (siRNA) targeting ISOC1 and a non-targeting siRNA control were purchased from Sangon Biotech (Shanghai, China). Transfection was performed using Lipo8000 transfection reagent (Beyotime, Shanghai, China) according to the manufacturer's instructions. After 48 hours of incubation, the efficiency of ISOC1 knockdown was confirmed by RT-qPCR. Cells were then subjected to downstream experiments.

### RNA isolation and quantitative real-time PCR (RT-qPCR)

Total RNA was extracted from cultured cells using the TRIzol reagent (Invitrogen, Carlsad, CA, USA) according to the manufacturer's instructions. cDNA was synthesized from 1µg of total RNA using the cDNA synthesis kit (Takara, Shiga, Japan). RT-qPCR was performed using TB Green Premix Ex Taq (Takara, Shiga, Japan) on a QuantStudio 5 RT-qPCR system (Thermo Fisher Scientific, USA). Gene expression was normalized to GAPDH and relative expression levels were calculated using the 2^-ΔΔCt^ method. Primer sequences are listed in Supplemental Table 1.

### Immunohistochemistry (IHC) staining

The ccRCC tumor samples and their corresponding adjacent normal tissue samples used in our study were obtained from surgical patients at the Department of Urology, Renmin Hospital of Wuhan University. All experiments were conducted in strict accordance with relevant ethical guidelines. Tissue samples were fixed with 4% paraformaldehyde and cut into 4-5 µm thick sections. After dewaxing and rehydration, the sections were washed with PBS. Endogenous peroxidase activity was blocked with 3% hydrogen peroxide, followed by blocking nonspecific binding sites with 5% BSA. The sections were incubated overnight at 4°C with ISOC1 antibody (mouse monoclonal, 1:50, Santa Cruz Biotechnology, Dallas, Texas, USA). The next day, the sections were washed with PBS and incubated with HRP-conjugated secondary antibody at room temperature for 1 hour. After staining by DAB chromogen, the protein expression was observed and analyzed under a microscope. Protein levels were quantified using ImageJ software.

### Transwell invasion assay

Cell invasion was assessed using Matrigel-coated Transwell inserts (8-μm pores, Corning, USA). 1 × 105 cells in serum-free medium were seeded into the upper chamber, with medium containing 10% FBS in the lower chamber as a chemoattractant. After 24 hours at 37°C, non-invasive cells were removed, and invaded cells on the lower membrane surface were fixed with 4% paraformaldehyde, stained with 0.1% crystal violet, and counted in five random fields under a microscope.

### Wound healing assay

Cells were seeded in 6-well plates and grown to nearly 100% confluence. A sterile pipette tip was used to create a scratch across the cell monolayer. The cells were washed with PBS to remove debris and then cultured in serum-free medium. Images of the wound area were captured at 0 hours and 24 hours using a light microscope.

### EdU (5-Ethynyl-2'-deoxyuridine) assay

Cell proliferation was assessed using the Click-iT EdU-594 Cell Proliferation Kit (Servicebio, Wuhan, China). Cells were seeded in 24-well plates and cultured to approximately 70-80% confluence. The cells were then incubated with EdU solution at a final concentration of 10 µM for 2 hours. After incubation, the cells were fixed with 4% paraformaldehyde for 15 minutes and washed with PBS to remove the fixative. The nuclei were stained with Hoechst dye. Finally, images were captured using a fluorescence microscope, and the percentage of EdU-positive cells was analyzed to assess cell proliferation.

### Statistical analysis

All statistical analyses were performed using the R software (4.4.1) and GraphPad Prism 8. Data are presented as mean ± standard deviation (SD) unless otherwise specified. Comparisons between two groups were conducted using the Student's t-test. All experiments were performed at least three times. Correlation analyses were conducted using Pearson or Spearman correlation coefficients, as appropriate. Statistical significance was set at P < 0.05.

## Results

### Data processing and acquisition of differentially expressed genes

**Figure 1:**
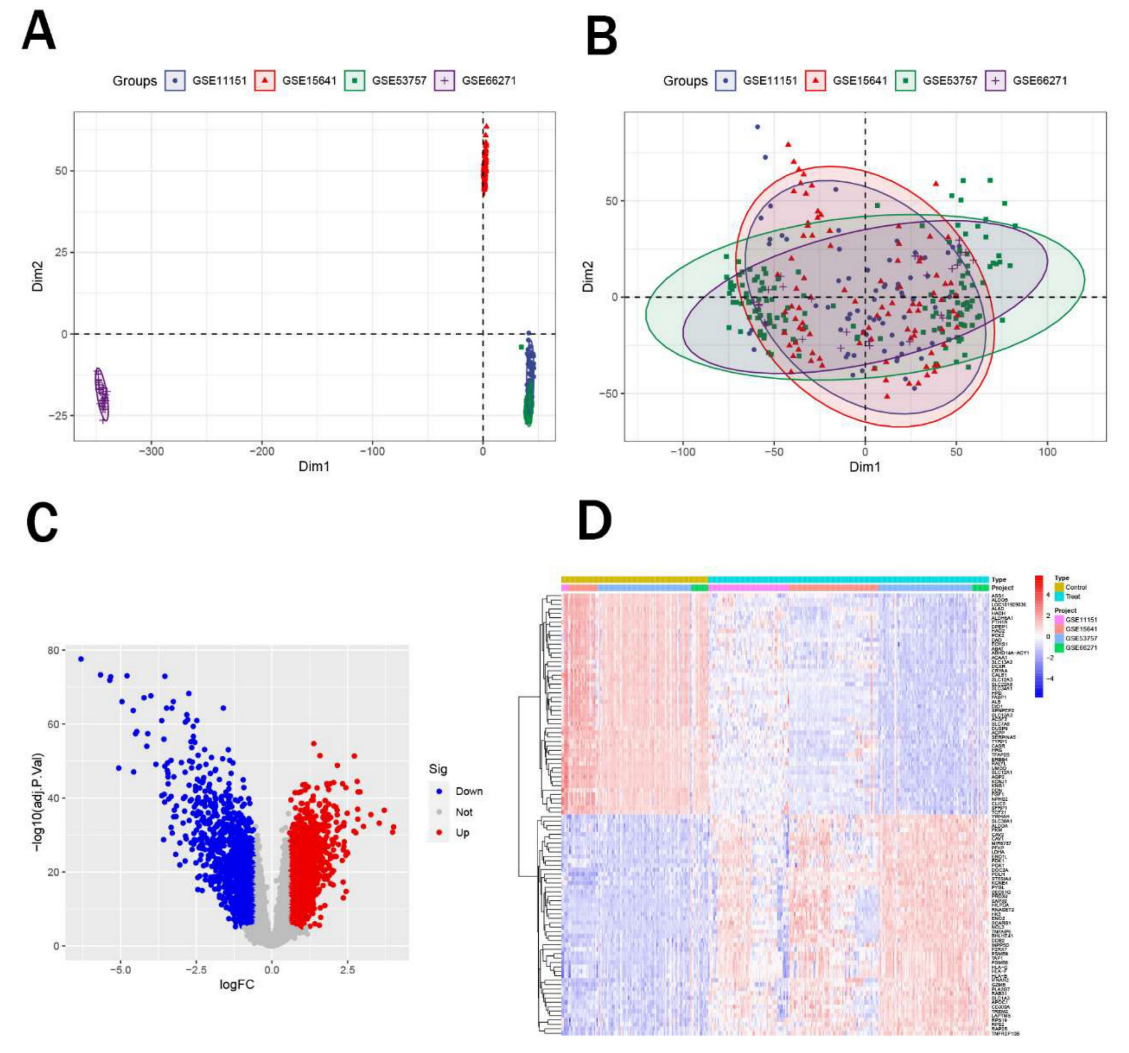
Identification of differentially expressed genes. (A-B) PCA plots showing sample distribution before (A) and after (B) batch effect correction across four renal cancer datasets. (C) Volcano plot of DEGs, with 1,228 upregulated (red) and 1,564 downregulated (blue) genes. (D) Heatmap of the top 50 significantly upregulated and downregulated genes.

Initially, we obtained five renal cancer datasets from the GEO database, with their detailed characteristics summarized in Table 1. Among these, GSE61441 was designated for subsequent external validation. Subsequently, we integrated the remaining four datasets (GSE11151, GSE15641, GSE53757, and GSE66271) and performed principal component analysis (PCA) to eliminate batch effects. Notably, after correction, samples from four datasets all showed enhanced uniformity (Figure 1A-B). Afterwards, we performed differential expression analysis on the merged data to identify differentially expressed genes. We identified a total of 1,228 upregulated genes and 1,564 downregulated genes (Figure 1C) and figure 1D displayed the top 50 significantly upregulated and downregulated genes.

### Mendelian randomization analysis and intersection genes

**Figure 2:**
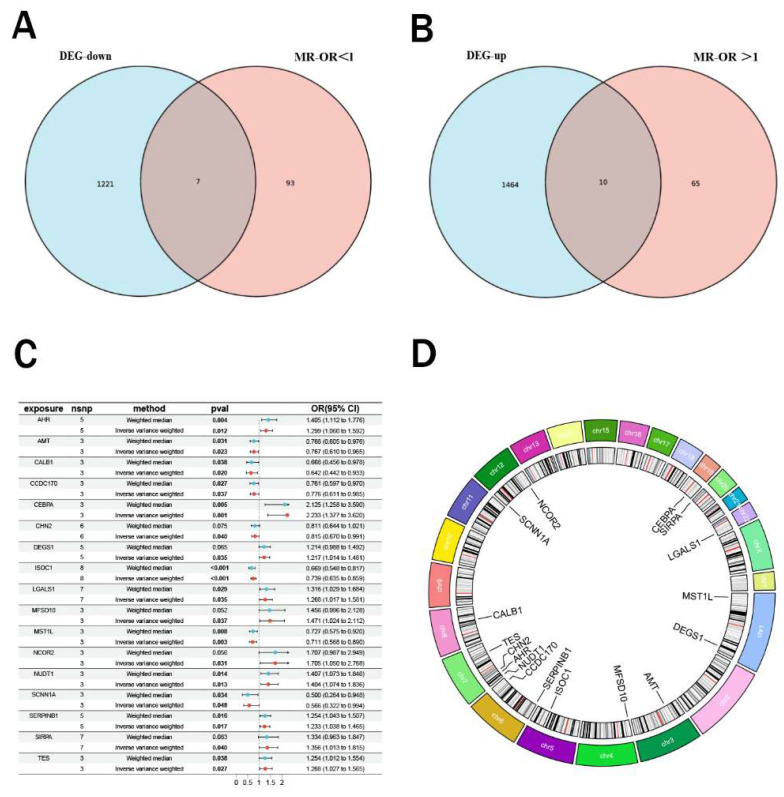
** Mendelian randomization analysis and intersection analysis.** (A-B) Venn diagram showing 17 intersection genes (10 upregulated, 7 downregulated) identified by intersecting 175 Mendelian randomization genes with DEGs. (C) Forest plot of odds ratios (ORs) indicating significant causal associations of all 17 genes with renal cancer (P < 0.05). (D) Chromosomal locations ofthe 17 intersection genes.

Using peripheral blood eQTL data of peripheral blood from the GWAS database and renal malignant tumor data from the Finnish cohort, we performed Mendelian randomization analysis. Based on the predefined screening criteria, we identified 175 genes associated with malignant neoplasm of kidney. By intersecting these with the differentially expressed genes, we identified 17 intersection genes, including 7 downregulated and 10 upregulated genes (Figure 2A-B). Subsequently, we performed Mendelian randomization analysis on these 17 genes to determine their individual effects on renal cancer. In the Mendelian randomization analysis using the IVW method, all 17 genes exhibited P- values less than 0.05, indicating a strong causal relationship with renal cancer. For the 10 upregulated genes, the odds ratios (OR) from all analysis methods were greater than 1, suggesting a significant positive causal association with renal cancer, while the downregulated genes showed the opposite trend (Figure 2C). We also conducted pleiotropy and heterogeneity tests, and the results showed P-values greater than 0.05 for all genes, indicating that pleiotropy and heterogeneity did not significantly impact the findings. Additionally, leave-one-out sensitivity analysis revealed that the effect sizes of individual SNP were close to the overall effect size, further confirming the robustness of our results. Detailed information on these 17 genes and the complete results of the Mendelian randomization analysis were provided in Table 2, with corresponding forest plots, scatter plots, and funnel plots in Supplementary Figure 1. Figure 2D illustrated the specific chromosomal locations of these 17 genes.

### Function enrichment analysis

To explore the potential biological functions of these 17 genes, we conducted GO and KGEE analyses. The GO analysis revealed that these genes are predominantly associated with functions closely related to gene transcription regulation and signal transduction, regulation of inflammatory response and immune cell activation (Supplemental Figure 1A). KEGG enrichment analysis indicated that these genes potentially involved in a range of key biological metabolic pathways, including folate metabolism, lipid biosynthesis and catabolism, as well as the synthesis and conversion of glyoxylate and dicarboxylates (Supplemental Figure 1B).

### pQTL Mendelian randomization

**Figure 3:**
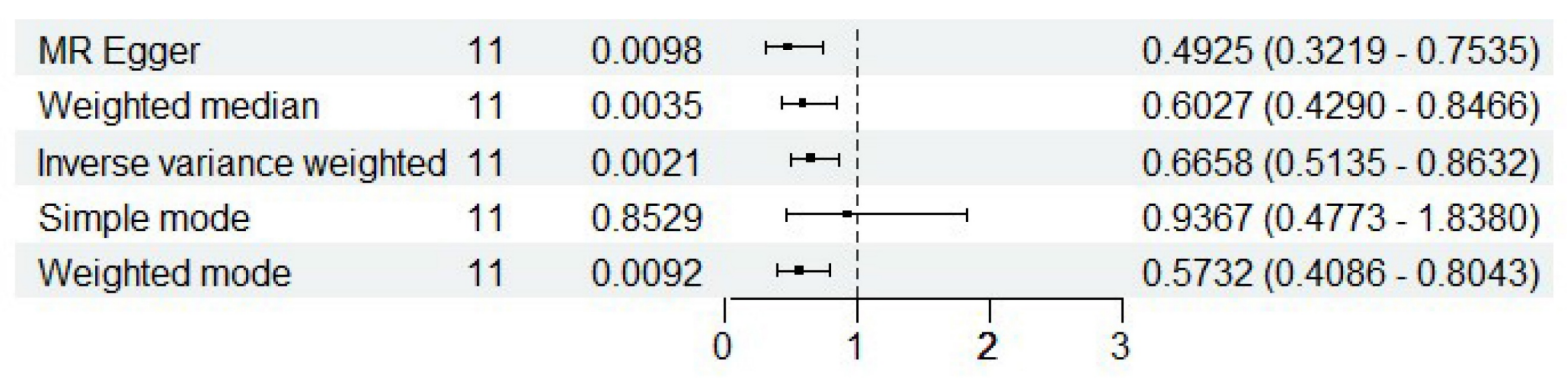
** pQTL Mendelian randomization analysis.** Analysis showed a negative causal relationship between ISOC1 protein levels and renal cancer, with P-values < 0.05 and odds ratios (OR) < 1 across four methods.

To systematically identify potential target genes for renal cancer, we conducted pQTL Mendelian randomization analysis on the 17 intersection genes previously identified, with the goal of elucidating the causal relationship between the protein levels of these genes and the onset of renal cancer. Due to the limited availability of pQTL data, we were unable to obtain pQTL information for some genes. Nevertheless, our findings demonstrate a robust causal association between the protein levels of ISOC1 and the development of renal cancer. As illustrated in Figure 3, the results from four distinct analytical approaches all yielded p-values below 0.05, with odds ratios (OR) consistently less than 1. This suggested a negative causal relationship between ISOC1 protein levels and renal cancer, aligning with our previous conclusions. Similarly, pleiotropy and heterogeneity tests and leave-one-out sensitivity analysis all ruled out the interference of other confounding factors on the conclusion. The corresponding forest plots, scatter plots, and funnel plots were provided in Supplementary Figure 2.

### The expression profile of ISOC1 in clear cell renal cell carcinoma

**Figure 4:**
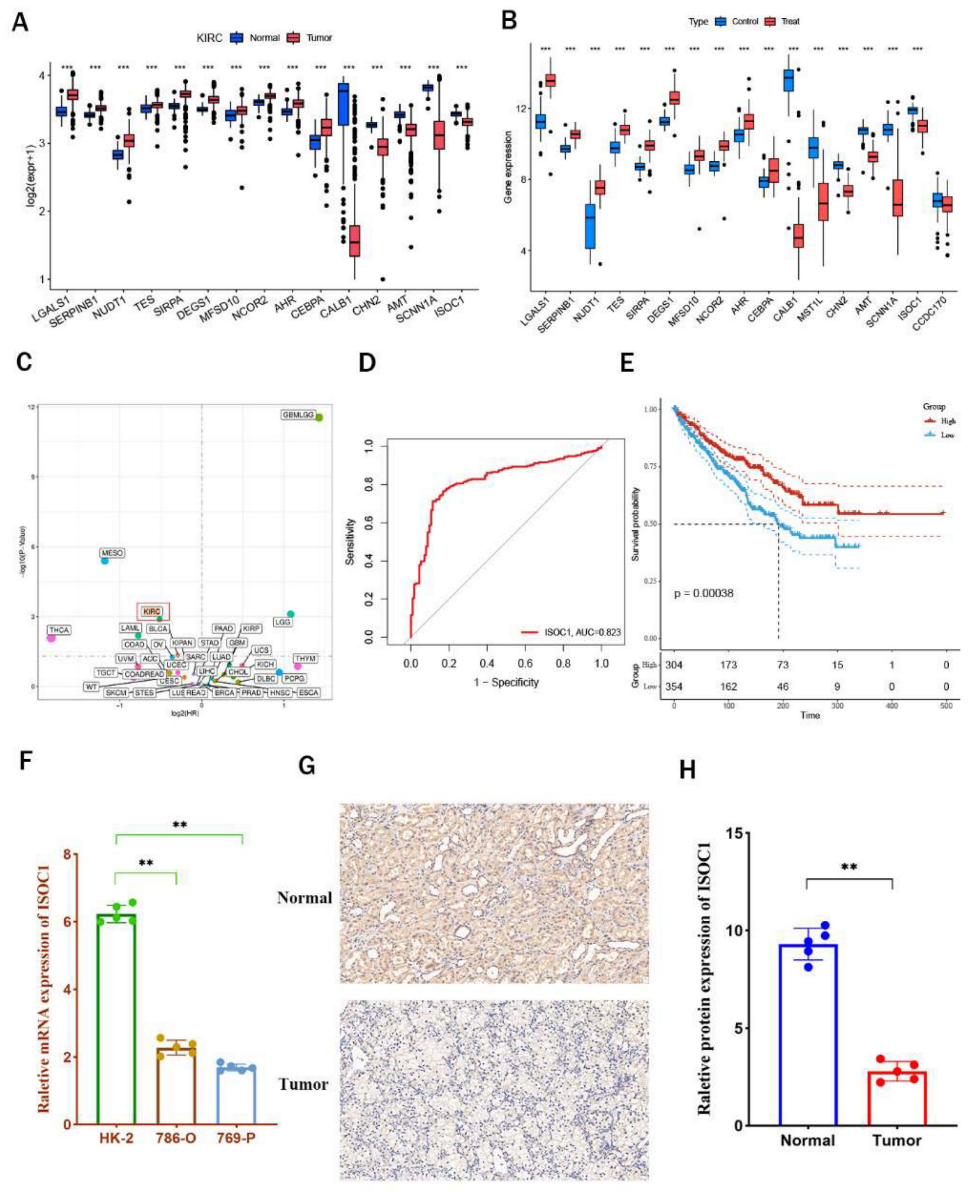
** ISOC1 expression and its prognostic value in clear cell renal cell carcinoma (ccRCC) (A-B)** Differential expression analysis of 17 co-expressed genes between RCC and adjacent normal tissues in the TCGA-KIRC and GSE61441 cohorts. (C) Pan-cancer analysis confirming ISOC1 as a protective factor for ccRCC. (D) ROC curve showing an AUC value of 0.823 for ISOC1 in predicting prognosis in the TCGA-KIBC cohort. (E) Survival analysis revealing significantly improved prognosis in high ISOC1 expression groups. (F) RT-qPCR showing higher ISOC1 expression in HK-2 cells compared to ccRCC cell lines. (G-H) Immunohistochemical staining demonstrating increased ISOC1 protein levels in adjacent normal tissues. *p < 0.05, **p < 0.01, ***p < 0.001.

We initially performed an analysis of the differential expression of 17 intersection genes between renal cancer and adjacent normal tissues using data from the TCGA-KIRC and GSE61441 cohorts (Figure 4A-B) and the results were all consistent with those obtained from eQTL Mendelian randomization analysis, Notably, ISOC1 was found to be highly expressed in adjacent normal tissues compared to tumor tissues. Subsequently, pan-cancer analysis further confirmed that ISOC1 acted as a protective factor for clear cell renal cell carcinoma (Figure 4C). The AUC value ofISOC1 for clear cell renal cell carcinoma was 0.823 in the TCGA-KIBC cohort, indicating that it could serve as a reliable predictor of prognosis (Figure 4D). Survival analysis revealed that individuals with high ISOC1 expression exhibited significantly improved prognosis compared to those with low ISOC1 expression (Figure 4E).

Subsequently, we performed in vitro experiments to further substantiate the expression ofISOC1 in clear cell renal cell carcinoma. RT-qPCR analysis revealed that ISOC1 expression was notably higher in HK-2 cells compared to the two clear cell renal cell carcinoma cell lines (Figure 4F). Additionally, immunohistochemical staining reinforced these observations, showing increased ISOC1 protein levels in adjacent normal tissues (Figure 4G-H), which was in alignment with our earlier analytical results. GSVA enrichment analysis indicated that the high-expression group of ISOC1 showed the most prominent enrichment in fatty acid metabolism, which is a key pathway in the formation and progression of ccRCC. In contrast, the low-expression group ofISOC1 exhibited the highest enrichment scores in the chemokine signaling pathway (Supplemental Figure 3).

### Immune cell infiltration and drug sensitivity

**Figure 5:**
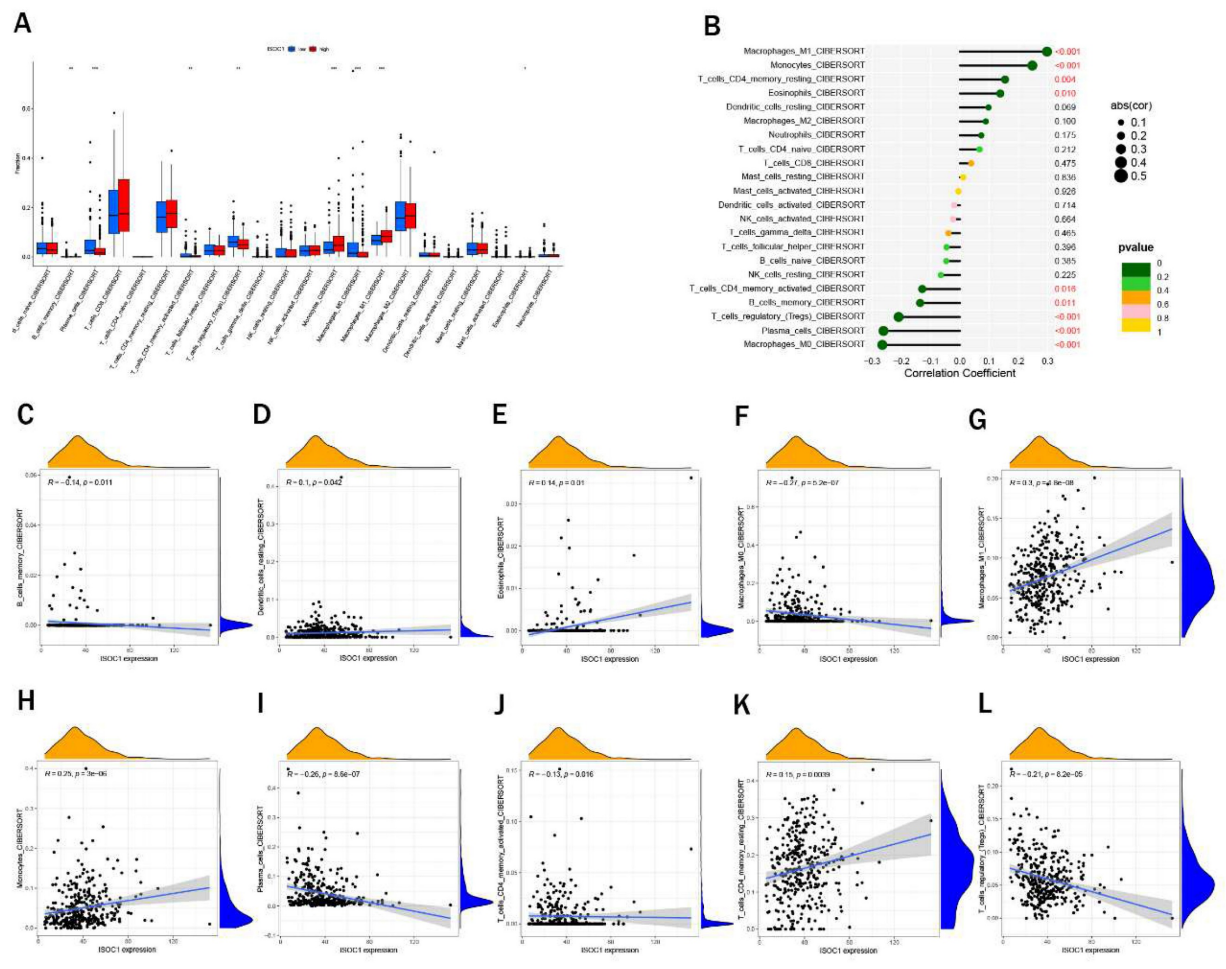
** Immune cell infiltration.** (A) Immune cell infiltration profiles in RCC cohorts with low or high ISOC1 expression using CIBERSORT. (B) Correlation between ISOC1 expression and immune cell infiltration in RCC. (C-L) Correlation scatter plots. *p < 0.05, **p < 0.01, ***p < 0.001.

**Figure 6:**
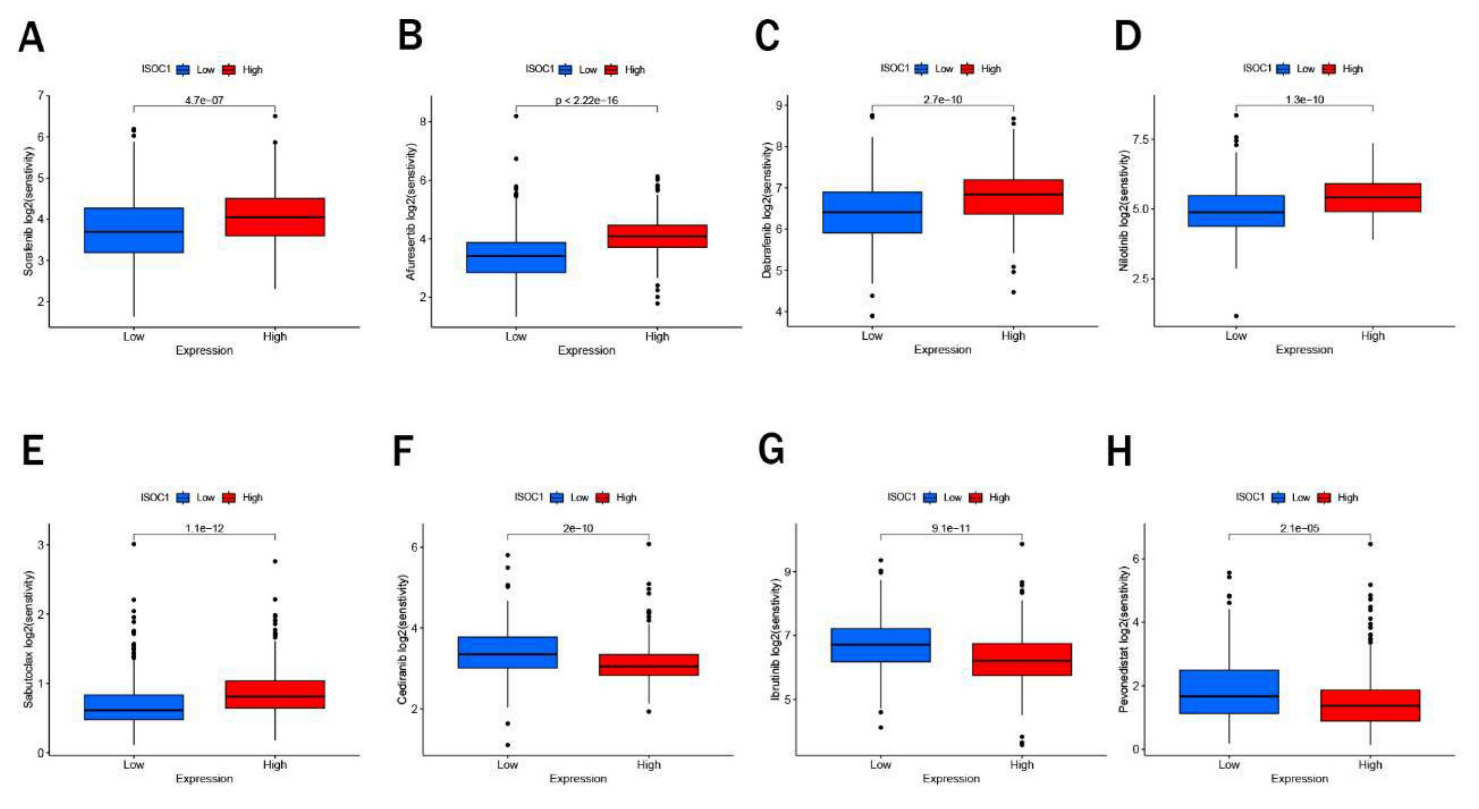
** Drug sensitivity.** (A-H) Sensitivity to targeted therapies and immunotherapies based on ISOC1 expression.

We employed the CIBERSORT algorithm to investigate the association between ISOCI and immune cell infiltration in renal cancer. We observed that lower expression ofISOC1 was strongly associated with a higher proportion of Macrophages M0, Plasma cells, T cells regulatory (Tregs) and B cells memory. In contrast, in the renal cancer cohort with high ISOC1 expression, Macrophages M1, Monocytes and Eosinophils appeared to be more activated (Figure 5A-B). Subsequent scatter plots further demonstrated the correlation between ISOC1 expression levels and the degree of immune cell infiltration. The infiltration levels of B memory cells, macrophage M0, plasma cells, T cells, CD4 memory activated and T cells regulatory (Tregs) showed a negative correlation with ISOC1 expression, whereas Dendritic cells resting, Eosinophils, Macrophages M1, Monocytes and T cells CD4 memory resting exhibited an opposite trend (Figure 5C-L). We also analyzed the association between ISOC1 expression levels and the sensitivity to common targeted therapies and immunotherapies in renal cancer cohorts. The analysis revealed that renal cancer patients with elevated ISOC1 expression demonstrated enhanced sensitivity to Sorafenib, Afuresertib, Dabrafenib, Nilotinib and Sabutoclax. Conversely, individuals with reduced ISOC1 expression appeared to have a more favorable response to Cediranib, Lbrutinib and Pevonedistat therapy (Figure 6).

### Knockdown of ISOC1 promoted the proliferation, migration, and invasion of renal cancer cells

**Figure 7:**
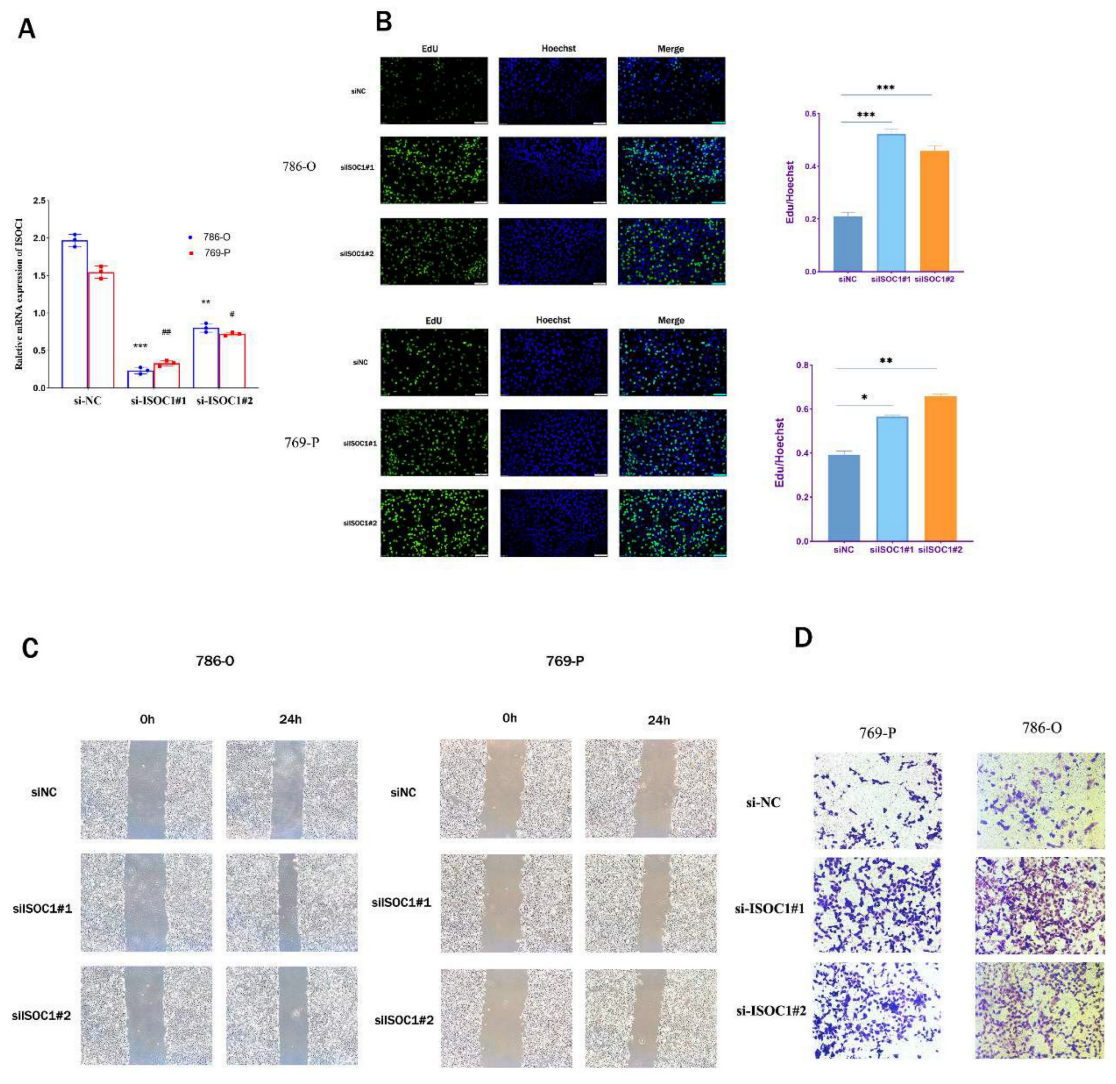
** Knockdown of ISOC1 promoted proliferation, migration, and invasion in ccRCC cells. (A)** RT-qPCR confirmed effective ISOC1 knockdown in 786-O and 769-P cells. (B) EdU assay showed increased cell proliferation in ISOC1 knockdown groups. (C) Transwell assay revealed enhanced invasion of RCC cells after ISOC1 knockdown. (D) Wound healing assay demonstrated increased migratory capacity in ISOC1-silenced cells. *p < 0.05, **p < 0.01, ***p < 0.001.

In order to further elucidate the role of ISOC1 in renal cancer, we undertook a series of in vitro experiments to explore its involvement in the progression and metastasis of RCC. Initially, we employed siRNA-mediated silencing of ISOC1 expression, followed by RT-qPCR to assess the knockdown efficiency. Clearly, the expression ofISOC1 was significantly reduced in both the 786- O and 769-P cell lines following siRNA treatment, compared to the control group (Figure 7A). The EdU assay was employed to assess the impact ofISOC1 on the proliferation of RCC cells. In both ISOC1 knockdown RCC cell lines, we observed a significant increase in both the number of cell colonies and the percentage of EdU-positive cells compared to the control group, indicating that ISOC1 downregulation enhances the proliferative capacity of renal cancer cells (Figure 7B). The Transwell assay confirmed that the inhibition of ISOC1 resulted in a reduced invasive ability of RCC cells (Figure 7C). The wound healing assay results demonstrated that ISOC1 promoted the migratory capacity of RCC cells (Figure 7D).

## Discussion

Renal cell carcinoma (RCC), particularly clear cell renal cell carcinoma (ccRCC), represents a significant challenge in oncology due to its insidious onset, high recurrence risk, and poor prognosis in advanced stages. In this study, we focused on identifying genetic and molecular mechanisms underlying RCC pathogenesis, with an emphasis on the role ofISOC1.

By employing Mendelian Randomization (MR) analyses that integrated eQTL and pQTL data, we identified a robust causal association between ISOC1 and RCC. This multidimensional approach, leveraging genetic, transcriptomic, and proteomic evidence, underscored the critical involvement of ISOC1 in RCC. This integrative approach also elucidates the biological pathways through which ISOC1 may contribute to RCC pathogenesis. For instance, the association ofISOC1 with immune cell infiltration and drug sensitivity underscores its multifaceted role in modulating both tumor behavior and the tumor microenvironment. Such findings are invaluable for identifying molecular targets with therapeutic potential. However, it is important to note some limitations of this methodology. While eQTL and pQTL analyses provide robust insights into gene and protein expression, they may not fully capture post-translational modifications or other layers of regulation influencing protein functionality^20^. Furthermore, the resolution of MR analyses depends on the quality and comprehensiveness of the genetic and phenotypic datasets used. Despite these limitations, the integration of eQTL and pQTL analyses within an MR framework offers a transformative approach for studying cancer biology^21^. In the context of RCC, this strategy has not only identified ISOC1 as a key player but also demonstrated the utility of combining genetic, transcriptomic, and proteomic data to uncover novel disease mechanisms. This approach holds promise for broader applications in precision oncology, facilitating the identification of biomarkers and therapeutic targets across diverse malignancies.

ISOC1 is a protein characterized by an isochorismatase-like domain. It can catalyze the conversion of isochorismate into 2,3-dihydroxy-2,3-dihydrobenzoate and pyruvate, suggesting its involvement in enzymatic processes^22^,^23^. Although its precise biological functions remain incompletely understood, existing evidence implicates ISOC1 in a range of cellular activities, particularly those related to metabolic regulation. Structurally, ISOC1 is hypothesized to play a role in modulating the synthesis and degradation of specific metabolic intermediates, yet its enzymatic activity and substrate specificity have not been conclusively defined^24^,^25^.

In the context of oncology, ISOC1 has garnered attention for its potential role in tumorigenesis^26^. Initial findings indicate that dysregulated ISOC1 expression may contribute to cancer-associated metabolic reprogramming, influencing cell proliferation and survival through alterations in metabolic pathways. For instance, changes in ISOC1 levels have been linked to shifts in cellular energy metabolism, thereby facilitating tumor growth and progression^27^-^29^. These observations suggest that ISOC1 may serve as a crucial mediator of metabolic and signaling networks in malignant transformation. The identification ofISOC1 through both eQTL and pQTL MR analyses underscored its robust genetic association with RCC. This integrative approach not only strengthened the validity of ISOC1 as a candidate gene, but also reflected the value of leveraging multi-omics MR strategies to uncover key regulators in cancer biology.

We performed GSVA enrichment analysis and found that high expression of ISOC1 was strongly associated with fatty acid metabolism. The link between fatty acid metabolism and ccRCC has garnered increasing attention in recent years^30^. Alterations in fatty acid metabolism are considered one of the key factors in tumor initiation and progression in ccRCC^31^,^32^. Tumor cells often undergo metabolic reprogramming to enhance fatty acid uptake, synthesis, and oxidation, adapting to the rapid growth and hypoxic microenvironment^33^,^34^. Moreover, fatty acid metabolism modulates immune cell infiltration and immune evasion, influencing the tumor microenvironment^35^. These findings highlight fatty acid metabolism as a promising therapeutic target in ccRCC.

Despite these advances, the precise molecular mechanisms and functional roles of ISOC1 remain largely unexplored. Notably, research on ISOC1 in renal cell carcinoma is currently lacking, leaving its role in this malignancy an open question for future investigation. Comprehensive studies are essential to elucidate its contributions to renal cancer pathogenesis and assess its potential as a biomarker or therapeutic target.

In conclusion, this study established ISOC1 as a key regulator in the pathogenesis and progression of RCC, illuminating its critical role in tumor biology. By employing MR-based integrative analyses, we provided a sophisticated framework for unraveling intricate oncogenic mechanisms. These findings underscored the potential of ISOC1 as a compelling biomarker and therapeutic target, laying a robust foundation for future advancements in RCC diagnostics and treatment strategies.

## Supplementary Material

Supplementary figures.

## Figures and Tables

**Table 1:** Summary of five GEO renal cancer datasets

GEO ID	Samples	Platform ID	Organism	Last update date	Experiment type	Tissues
GSE11151	5 controls and 62 tumors	GPL570	Homo sapiens	8-Aug-24	Array	Kidney tissues
GSE15641	23 controls and 69 tumors	GPL96	Homo sapiens	10-Aug-18	Array	Kidney tissues
GSE53757	72 controls and 72 tumors	GPL570	Homo sapiens	25-Mar-19	Array	Kidney tissues
GSE66271	13 controls and 13 tumors	GPL570	Homo sapiens	24-Jul-19	Array	Kidney tissues
GSE61441	46 controls and 46 tumors	GPL13534	Homo sapiens	22-Mar-19	Array	Kidney tissues

**Table 2:** Specific eQTL MR analysis results of the 17 intersection genes

id.exposure	id.outcom outcome	exposure method	nsnp		b	se	pval	lo_ci	up_ci	or	or_lci95	or_uci95
eqtl-a-ENSG00000 12CK9u	Malignant neoplasm of kidney	AHR	MR Egger	5	0.349773	0.16152	0.118956	0.033195	0.666352	1.418746	1.033752	1.947121
eqtl-a-ENSG00000 12CK9u	Malignant neoplasm of kidney	AHR	Weighted	5	0.340134	0.119582	0.00445	0.105752	0.574515	1.405135	1.111547	1.776269
eqtl-a-ENSG00000 12CK9u	Malignant neoplasm of kidney	AHR	Inverse va	5	0.261444	0.10383	0.011802	0.057937	0.46495	1.298804	1.059649	1.591935
eqtl-a-ENSG00000 12CK9u	Malignant neoplasm of kidney	AHR	Simple m	5	0.332979	0.196882	0.166047	-0.05291	0.718869	1.395119	0.948465	2.05211
eqtl-a-ENSG00000 12CK9u	Malignant neoplasm of kidney	AHR	Weighted	5	0.359505	0.131335	0.05205	0.102088	0.616923	1.432621	1.107481	1.853216
eqtl-a-ENSG00000 9D8ME4	Malignant neoplasm of kidney	AMT	MR Egger	3	-0.37196	0.346433	0.477389	-1.05097	0.307049	0.689382	0.349599	1.359408
eqtl-a-ENSG00000 9D8ME4	Malignant neoplasm of kidney	AMT	Weighted	3	-0.26336	0.1219	0.030738	-0.50228	-0.02443	0.768467	0.605148	0.975862
eqtl-a-ENSG00000 9D8ME4	Malignant neoplasm of kidney	AMT	Inverse va	3	-0.26496	0.11688	0.023396	-0.49404	-0.03587	0.76724	0.610156	0.964765
eqtl-a-ENSG00000 9D8ME4	Malignant neoplasm of kidney	AMT	Simple m	3	-0.1887	0.175061	0.393811	-0.53182	0.15442	0.828036	0.587536	1.166981
eqtl-a-ENSG00000 9D8ME4	Malignant neoplasm of kidney	AMT	Weighted	3	-0.28318	0.121407	0.144895	-0.52114	-0.04522	0.753382	0.593843	0.955782
eqtl-a-ENSG00000 zlrvEZ	Malignant neoplasm of kidney	CALB1	MR Egger	3	-0.1062	1.106795	0.939101	-2.27552	2.063118	0.899245	0.102744	7.870471
eqtl-a-ENSG00000 zlrvEZ	Malignant neoplasm of kidney	CALB1	Weighted	3	-0.40344	0.194294	0.037853	-0.78426	-0.02262	0.668018	0.456459	0.97763
eqtl-a-ENSG00000 zlrvEZ	Malignant neoplasm of kidney	CALB1	Inverse va	3	-0.44308	0.190578	0.020077	-0.81661	-0.06954	0.642058	0.441928	0.93282
eqtl-a-ENSG00000 zlrvEZ	Malignant neoplasm of kidney	CALB1	Simple m	3	-0.40764	0.274806	0.27622	-0.94626	0.130977	0.665216	0.388189	1.139941
eqtl-a-ENSG00000 zlrvEZ	Malignant neoplasm of kidney	CALB1	Weighted	3	-0.39882	0.220097	0.211673	-0.83021	0.032568	0.671111	0.435957	1.033104
eqtl-a-ENSG00000 q8Ydpb	Malignant neoplasm of kidney	CCDC170	MR Egger	3	-0.4584	0.19237	0.252952	-0.83544	-0.08135	0.632296	0.433682	0.921867
eqtl-a-ENSG00000 q8Ydpb	Malignant neoplasm of kidney	CCDC170	Weighted	3	-0.27339	0.123832	0.02726	-0.5161	-0.03068	0.760795	0.596843	0.969784
eqtl-a-ENSG00000 q8Ydpb	Malignant neoplasm of kidney	CCDC170	Inverse va	3	-0.25401	0.122007	0.037348	-0.49314	-0.01488	0.775683	0.610703	0.985232
eqtl-a-ENSG00000 q8Ydpb	Malignant neoplasm of kidney	CCDC170	Simple m	3	-0.25273	0.175144	0.285805	-0.59602	0.09055	0.776675	0.551002	1.094776
eqtl-a-ENSG00000 q8Ydpb	Malignant neoplasm of kidney	CCDC170	Weighted	3	-0.28553	0.136483	0.171537	-0.55303	-0.01802	0.751619	0.575203	0.982143
eqtl-a-ENSG00000ylnzDz	Malignant neoplasm of kidney	CEBPA	MR Egger	3	0.403433	0.847201	0.717071	-1.25708	2.063946	1.496955	0.284483	7.876991
eqtl-a-ENSG00000ylnzDz	Malignant neoplasm of kidney	CEBPA	Weighted	3	0.753704	0.267544	0.004846	0.229317	1.278092	2.124857	1.257741	3.589783
eqtl-a-ENSG00000ylnzDz	Malignant neoplasm of kidney	CEBPA	Inverse va	3	0.803187	0.246591	0.001125	0.319868	1.286506	2.232645	1.376946	3.620118
eqtl-a-ENSG00000ylnzDz	Malignant neoplasm of kidney	CEBPA	Simple m	3	0.711711	0.337666	0.1696	0.049886	1.373536	2.037475	1.051152	3.949291
eqtl-a-ENSG00000ylnzDz	Malignant neoplasm of kidney	CEBPA	Weighted	3	0.732443	0.317208	0.147235	0.110715	1.354172	2.080156	1.117076	3.873551
eqtl-a-ENSG00000 sKm18l	Malignant neoplasm of kidney	CHN2	MR Egger	6	-0.24864	0.153882	0.181449	-0.55025	0.052971	0.779863	0.576808	1.0544
eqtl-a-ENSG00000 sKm18l	Malignant neoplasm of kidney	CHN2	Weighted	6	-0.20943	0.117526	0.07475	-0.43978	0.02092	0.811046	0.644177	1.02114
eqtl-a-ENSG00000 sKm18l	Malignant neoplasm of kidney	CHN2	Inverse va	6	-0.20505	0.099854	0.040027	-0.40076	-0.00933	0.814609	0.66981	0.99071
eqtl-a-ENSG00000 sKm18l	Malignant neoplasm of kidney	CHN2	Simple m	6	-0.33625	0.206383	0.164191	-0.74076	0.068263	0.714447	0.476753	1.070647
eqtl-a-ENSG00000 sKm18l	Malignant neoplasm of kidney	CHN2	Weighted	6	-0.19173	0.122196	0.177435	-0.43123	0.047776	0.825531	0.649707	1.048936
eqtl-a-ENSG00000 QqATFT	Malignant neoplasm of kidney	DEGS1	MR Egger	5	0.162062	0.167412	0.404429	-0.16607	0.490189	1.175933	0.846991	1.632625
eqtl-a-ENSG00000 QqATFT	Malignant neoplasm of kidney	DEGS1	Weighted	5	0.194207	0.105083	0.064582	-0.01175	0.400169	1.214348	0.988314	1.492077
eqtl-a-ENSG00000 QqATFT	Malignant neoplasm of kidney	DEGS1	Inverse va	5	0.196545	0.09309	0.034743	0.014088	0.379001	1.21719	1.014188	1.460824
eqtl-a-ENSG00000 QqATFT	Malignant neoplasm of kidney	DEGS1	Simple m	5	0.263013	0.148997	0.152287	-0.02902	0.555047	1.300843	0.971395	1.742023
eqtl-a-ENSG00000 QqATFT	Malignant neoplasm of kidney	DEGS1	Weighted	5	0.186776	0.116016	0.182704	-0.04062	0.414168	1.205357	0.960198	1.513111
eqtl-a-ENSG00000 k9zPiw	Malignant neoplasm of kidney	ISOC1	MR Egger	8	-0.42557	0.191175	0.067628	-0.80028	-0.05087	0.653395	0.449205	0.950402
eqtl-a-ENSG00000 k9zPiw	Malignant neoplasm of kidney	ISOC1	Weighted	8	-0.40207	0.102137	8.26E-05	-0.60226	-0.20188	0.668934	0.547574	0.817191
eqtl-a-ENSG00000 k9zPiw	Malignant neoplasm of kidney	ISOC1	Inverse va	8	-0.30302	0.077223	8.71E-05	-0.45437	-0.15166	0.738586	0.634845	0.859279
eqtl-a-ENSG00000 k9zPiw	Malignant neoplasm of kidney	ISOC1	Simple m	8	-0.40771	0.150757	0.030444	-0.70319	-0.11223	0.665172	0.495002	0.893842
eqtl-a-ENSG00000 k9zPiw	Malignant neoplasm of kidney	ISOC1	Weighted	8	-0.4044	0.098116	0.00445	-0.59671	-0.21209	0.667377	0.550622	0.808891
eqtl-a-ENSG00000 7RymRD	Malignant neoplasm of kidney	LGALS1	MR Egger	7	0.316633	0.164075	0.111518	-0.00495	0.638221	1.372499	0.995058	1.89311
eqtl-a-ENSG00000 7RymRD	Malignant neoplasm of kidney	LGALS1	Weighted	7	0.274717	0.125764	0.028933	0.02822	0.521214	1.316158	1.028622	1.68407
eqtl-a-ENSG00000 7RymRD	Malignant neoplasm of kidney	LGALS1	Inverse va	7	0.237328	0.112545	0.034967	0.01674	0.457916	1.267857	1.016881	1.580776
eqtl-a-ENSG00000 7RymRD	Malignant neoplasm of kidney	LGALS1	Simple m	7	0.204201	0.235664	0.419517	-0.2577	0.666103	1.226545	0.772827	1.946637
eqtl-a-ENSG00000 7RymRD	Malignant neoplasm of kidney	LGALS1	Weighted	7	0.270783	0.134202	0.090183	0.007748	0.533819	1.310991	1.007778	1.705433
eqtl-a-ENSG00000 MFtVet	Malignant neoplasm of kidney	MFSD10	MR Egger	3	0.036844	0.485049	0.951736	-0.91385	0.987539	1.037531	0.400977	2.68462
eqtl-a-ENSG00000 MFtVet	Malignant neoplasm of kidney	MFSD10	Weighted	3	0.375514	0.19362	0.052448	-0.00398	0.755009	1.455739	0.996027	2.12763
eqtl-a-ENSG00000 MFtVet	Malignant neoplasm of kidney	MFSD10	Inverse va	3	0.385929	0.184593	0.036555	0.024126	0.747731	1.47098	1.02442	2.112202
eqtl-a-ENSG00000 MFtVet	Malignant neoplasm of kidney	MFSD10	Simple m	3	0.847153	0.38246	0.157143	0.097531	1.596776	2.332996	1.102446	4.937088
eqtl-a-ENSG00000 MFtVet	Malignant neoplasm of kidney	MFSD10	Weighted	3	0.329775	0.198096	0.237879	-0.05849	0.718042	1.390655	0.943186	2.050415
eqtl-a-ENSG00000 VbwQ5C	Malignant neoplasm of kidney	MST1L	MR Egger	3	-0.21642	0.513604	0.746118	-1.22308	0.790243	0.805397	0.294321	2.203932
eqtl-a-ENSG00000 VbwQ5C	Malignant neoplasm of kidney	MST1L	Weighted	3	-0.31862	0.119929	0.00789	-0.55368	-0.08356	0.72715	0.574828	0.919835
eqtl-a-ENSG00000 VbwQ5C	Malignant neoplasm of kidney	MST1L	Inverse va	3	-0.34108	0.114664	0.002934	-0.56582	-0.11633	0.711004	0.567895	0.890177
eqtl-a-ENSG00000 VbwQ5C	Malignant neoplasm of kidney	MST1L	Simple m	3	-0.33519	0.15748	0.167093	-0.64385	-0.02653	0.715204	0.525268	0.973822
eqtl-a-ENSG00000 VbwQ5C	Malignant neoplasm of kidney	MST1L	Weighted	3	-0.31493	0.114576	0.110795	-0.5395	-0.09036	0.729842	0.583043	0.913604
eqtl-a-ENSG00000 lGGMY6	Malignant neoplasm of kidney	NCOR2	MR Egger	3	0.951572	0.96941	0.505911	-0.94847	2.851615	2.589778	0.387333	17.31573
eqtl-a-ENSG00000 lGGMY6	Malignant neoplasm of kidney	NCOR2	Weighted	3	0.534456	0.279122	0.055521	-0.01262	1.081534	1.706519	0.987456	2.949201
eqtl-a-ENSG00000 lGGMY6	Malignant neoplasm of kidney	NCOR2	Inverse va	3	0.533493	0.247254	0.030953	0.048876	1.018111	1.704878	1.05009	2.767962
eqtl-a-ENSG00000 lGGMY6	Malignant neoplasm of kidney	NCOR2	Simple m	3	0.422374	0.330033	0.329009	-0.22449	1.069238	1.525579	0.798924	2.913159
eqtl-a-ENSG00000 lGGMY6	Malignant neoplasm of kidney	NCOR2	Weighted	3	0.547228	0.299701	0.209403	-0.04019	1.134643	1.728456	0.960611	3.110063
eqtl-a-ENSG00000 qr9bM4	Malignant neoplasm of kidney	NUDT1	MR Egger	3	0.443846	0.281634	0.35996	-0.10816	0.995848	1.558691	0.897488	2.707019
eqtl-a-ENSG00000 qr9bM4	Malignant neoplasm of kidney	NUDT1	Weighted	3	0.341755	0.138436	0.013561	0.070421	0.61309	1.407416	1.072959	1.846127
eqtl-a-ENSG00000 qr9bM4	Malignant neoplasm of kidney	NUDT1	Inverse va	3	0.339472	0.136799	0.013081	0.071346	0.607598	1.404206	1.073953	1.836015
eqtl-a-ENSG00000 qr9bM4	Malignant neoplasm of kidney	NUDT1	Simple m	3	0.159256	0.238764	0.573423	-0.30872	0.627234	1.172638	0.734385	1.872425
eqtl-a-ENSG00000 qr9bM4	Malignant neoplasm of kidney	NUDT1	Weighted	3	0.358488	0.145988	0.133436	0.072351	0.644625	1.431164	1.075033	1.905273
eqtl-a-ENSG00000 9Ir75v	Malignant neoplasm of kidney	SCNN1A	MR Egger	3	-1.22319	1.05292	0.452466	-3.28691	0.840537	0.294291	0.037369	2.317612
eqtl-a-ENSG00000 9Ir75v	Malignant neoplasm of kidney	SCNN1A	Weighted	3	-0.6931	0.3262	0.033606	-1.33245	-0.05375	0.500023	0.263829	0.94767
eqtl-a-ENSG00000 9Ir75v	Malignant neoplasm of kidney	SCNN1A	Inverse va	3	-0.5696	0.287489	0.047558	-1.13308	-0.00612	0.56575	0.322039	0.993895
eqtl-a-ENSG00000 9Ir75v	Malignant neoplasm of kidney	SCNN1A	Simple m	3	-0.7713	0.465773	0.239569	-1.68422	0.141614	0.462411	0.18559	1.152132
eqtl-a-ENSG00000 9Ir75v	Malignant neoplasm of kidney	SCNN1A	Weighted	3	-0.72718	0.372376	0.19008	-1.45704	0.00268	0.483271	0.232926	1.002683
eqtl-a-ENSG00000 GQ85n7	Malignant neoplasm of kidney	SERPINB1	MR Egger	5	0.209544	0.13757	0.225094	-0.06009	0.479181	1.233115	0.941676	1.614752
eqtl-a-ENSG00000 GQ85n7	Malignant neoplasm of kidney	SERPINB1	Weighted	5	0.226284	0.093881	0.015938	0.042278	0.410291	1.253932	1.043184	1.507256
eqtl-a-ENSG00000 GQ85n7	Malignant neoplasm of kidney	SERPINB1	Inverse va	5	0.209342	0.087989	0.017351	0.036884	0.3818	1.232867	1.037573	1.464919
eqtl-a-ENSG00000 GQ85n7	Malignant neoplasm of kidney	SERPINB1	Simple m	5	0.202517	0.175045	0.311682	-0.14057	0.545606	1.224481	0.868862	1.725654
eqtl-a-ENSG00000 GQ85n7	Malignant neoplasm of kidney	SERPINB1	Weighted	5	0.234177	0.097911	0.075027	0.042271	0.426083	1.263868	1.043177	1.531247
eqtl-a-ENSG00000 SmFZJz	Malignant neoplasm of kidney	SIRPA	MR Egger	7	0.009615	0.338833	0.97846	-0.6545	0.673726	1.009661	0.519703	1.961533
eqtl-a-ENSG00000 SmFZJz	Malignant neoplasm of kidney	SIRPA	Weighted	7	0.288183	0.166005	0.082566	-0.03719	0.613553	1.334001	0.963496	1.846982
eqtl-a-ENSG00000 SmFZJz	Malignant neoplasm of kidney	SIRPA	Inverse va	7	0.304636	0.148604	0.040365	0.013373	0.5959	1.356132	1.013463	1.814663
eqtl-a-ENSG00000 SmFZJz	Malignant neoplasm of kidney	SIRPA	Simple m	7	0.079502	0.278155	0.784627	-0.46568	0.624687	1.082748	0.627707	1.867661
eqtl-a-ENSG00000 SmFZJz	Malignant neoplasm of kidney	SIRPA	Weighted	7	0.283689	0.179803	0.165691	-0.06872	0.636102	1.32802	0.933584	1.889103
eqtl-a-ENSG00000 pe7YcH	Malignant neoplasm of kidney	TES	MR Egger	3	0.063144	0.290703	0.863835	-0.50663	0.632922	1.06518	0.60252	1.883105
eqtl-a-ENSG00000 pe7YcH	Malignant neoplasm of kidney	TES	Weighted	3	0.226342	0.109346	0.038456	0.012024	0.440659	1.254004	1.012097	1.553731
eqtl-a-ENSG00000 pe7YcH	Malignant neoplasm of kidney	TES	Inverse va	3	0.237309	0.107496	0.027272	0.026617	0.448	1.267832	1.026974	1.565179
eqtl-a-ENSG00000 pe7YcH	Malignant neoplasm of kidney	TES	Simple m	3	0.153909	0.17549	0.472968	-0.19005	0.497869	1.166384	0.826917	1.645211
eqtl-a-ENSG00000 pe7YcH	Malignant neoplasm of kidney	TES	Weighted	3	0.208419	0.115275	0.212336	-0.01752	0.434358	1.231729	0.982632	1.543971
