# Supplementary material for: Integrative Analysis of GEO Datasets and Mendelian Randomization Reveals a Potential Role ofISOC1 in Renal Cell Carcinoma
Source: J Cancer. 2025 Oct 10;16(14):4219–32. doi: 10.7150/jca.118622 (PMC12595268; doi:10.7150/jca.118622)
Supplement: Supplementary file 1 — Supplementary figures. [file jcav16p4219s1.pdf]

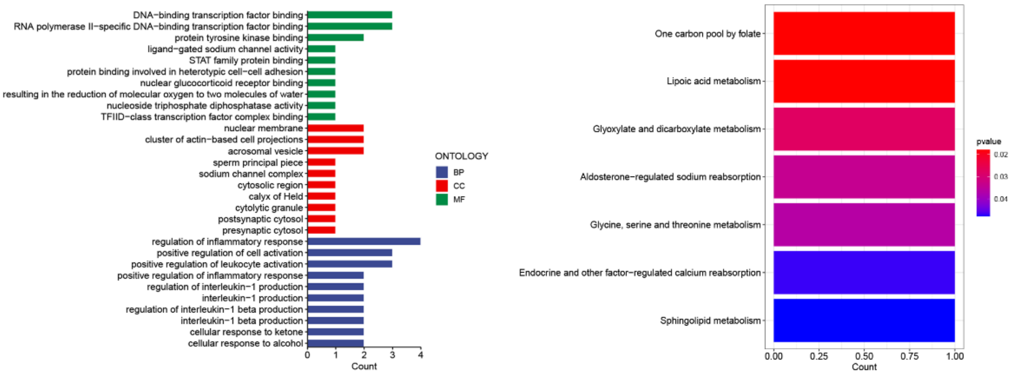

Supplemental Figure 1 GO (A) and KEGG (B) enrichment analysis of the 17 intersection genes.

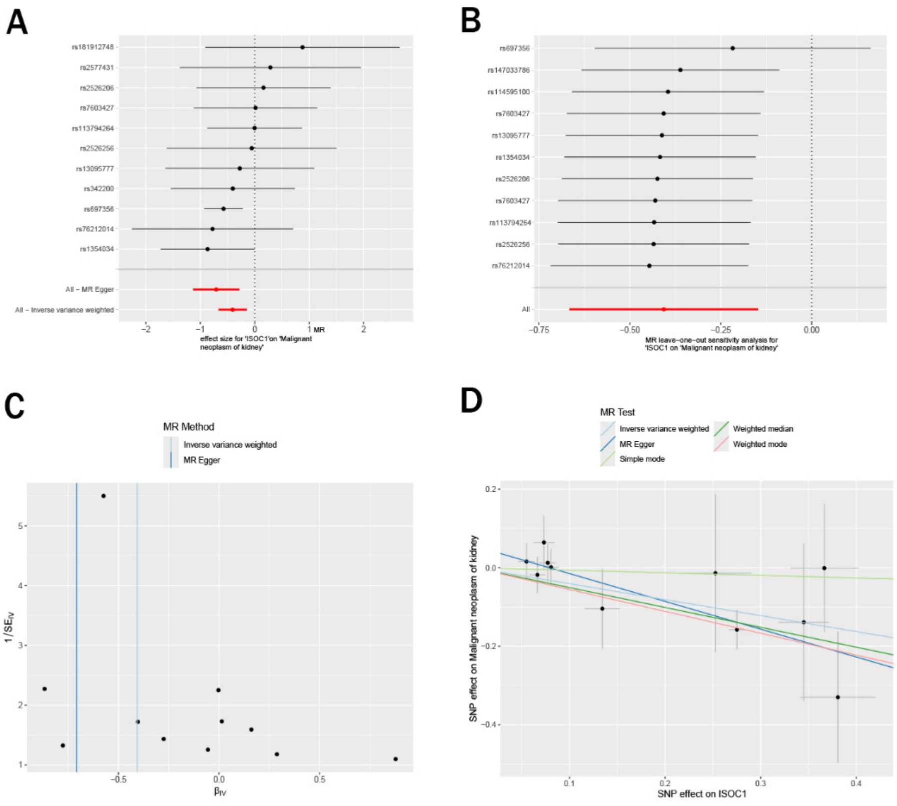

Supplemental Figure 2 Forest plots, leave-one-out sensitivity analysis plots, funnel plots and scatterplots ofISOC1 for pQTL MR analysis.

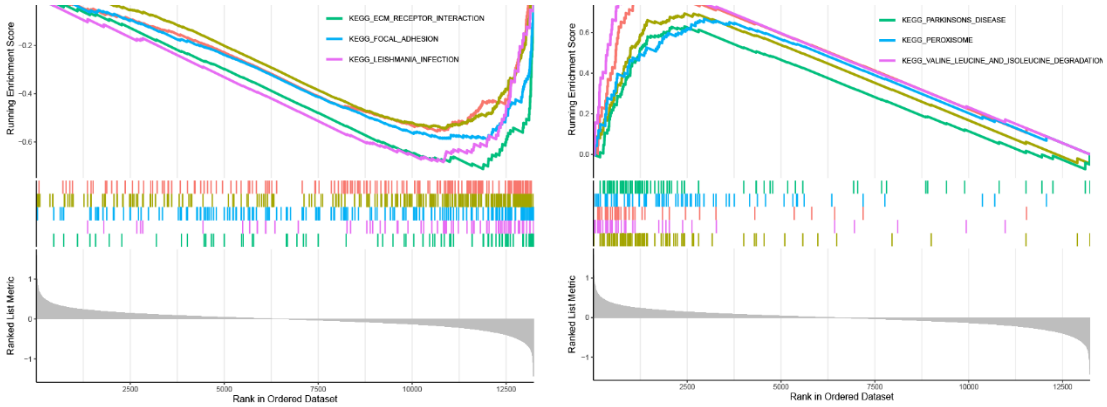

Supplemental Figure 3 GSVA enrichment analysis for ISOC1 in RCC.

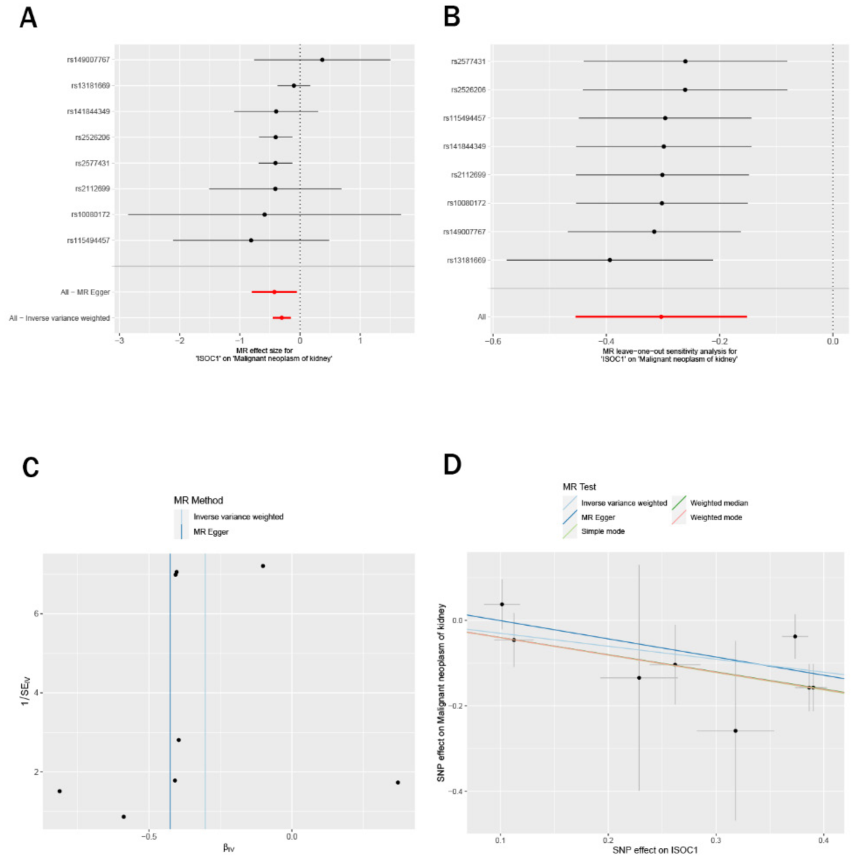

Supplemental Figure 4 Forest plots, leave-one-out sensitivity analysis plots, funnel plots and

scatterplots ofISOC1 for eQTL MR analysis.
